# The impact of DNA tumor viruses in low-to-middle income countries (LMICS): A literature review

**DOI:** 10.1016/j.tvr.2024.200289

**Published:** 2024-07-06

**Authors:** Bothwell Takaingofa Guzha, Allen Matubu, George Nyandoro, Hamish O. Mubata, Enos Moyo, Grant Murewanhema, Zvavahera M. Chirenje

**Affiliations:** a Department of Obstetrics and Gynaecology, Faculty of Medicine and Health Sciences, University of Zimbabwe, Harare, Zimbabwe; b University of Zimbabwe Clinical Trials Research Centre, Harare, Zimbabwe; c Hepatitis Alliance, 2172, Arlington, Hatfield, Harare, Zimbabwe; d School of Public Health Medicine, College of Health Sciences, University of KwaZulu-Natal, Durban, South Africa; e Department of Obstetrics, Gynecology and Reproductive Science, University of California San Francisco, San Francisco, USA

**Keywords:** DNA tumor viruses, HPV, HBV, EBV, Kaposi sarcoma, Polyoma

## Abstract

DNA viruses are common in the human population and act as aetiological agents of cancer on a large scale globally. They include the human papillomaviruses (HPV), Epstein-Barr virus (EBV), Kaposi sarcoma-associated herpesvirus (KSHV), hepatitis viruses, and human polyomaviruses. Oncogenic viruses employ different mechanisms to induce cancer. Notably, cancer only develops in a minority of individuals who are infected, usually following protracted years of chronic infection. The human papillomaviruses (HPVs) are associated with the highest number of cancer cases, including cervical cancer and other epithelial malignancies. Hepatitis B virus (HBV) and the RNA virus hepatitis C (HCV) are significant contributors to hepatocellular cancer (HCC). Other oncoviruses include Epstein-Barr virus (EBV), Kaposi sarcoma-associated herpes virus (KSHV), human T-cell leukemia virus (HTLV-I), and Merkel cell polyomavirus (MCPyV). The identification of these infectious agents as aetiological agents for cancer has led to reductions in cancer incidence through preventive interventions such as HBV and HPV vaccination, HPV-DNA based cervical cancer screening, antiviral treatments for chronic HBV and HCV infections, and screening of blood for transfusion for HBV and HCV. Successful efforts to identify additional oncogenic viruses in human cancer may provide further understanding of the aetiology and development of cancer, and novel approaches for prevention and treatment. Cervical cancer, caused by HPV, is the leading gynaecological malignancy in LMICs, with high age-standardised incidence and mortality rates, HCC due to HBV is an important cause of cancer deaths, and the burden of other cancer attributable to infections continues to rise globally. Hence, cancers attributable to DNA viruses have become a significant global health challenge. These viruses hence warrant continued attention and interrogation as efforts to understand them further and device further preventive interventions are critical.

## 1 Introduction

Deoxyribonucleic acid (DNA) tumor viruses are a group of viruses that can induce cancer in both humans and animals. They are referred to as “tumor viruses” due to their ability to trigger uncontrolled cell division, resulting in the development of tumors [1]. The initial DNA tumor viruses to be identified were the rabbit fibroma virus and the Shope papilloma virus, both of which were discovered by Richard Shope during the 1930s [2]. Since then, numerous other DNA tumor viruses have been identified, including the human papillomavirus (HPV), hepatitis B virus (HBV), and Epstein-Barr virus (EBV) [1]. These viruses have the potential to cause cancers such as cervical cancer, liver cancer, and Burkitt's lymphoma [1].

This review highlights the significant impact of DNA tumor viruses on the fields of molecular biology and cancer research in low-to-middle income countries (LMICs). This information helps researchers by synthesizing, consolidating, and summarizing publications from all over the world to enable discussions on the latest findings and current theories that shape our understanding of this diverse group of viruses and their impact on human health. About 70 % of cancer deaths occur in LMICs; this represents a disproportionately large burden, with 20 % of cancer-attributable deaths reported globally [3]. The causes of the disparities in cancer deaths between high-income countries (HICs) and LMICs are multifactorial. These include environmental, occupational and lifestyle factors. Molecular variations resulting from environmental exposures, genetic factors, and regional habits may contribute to the development of cancer and require further investigation [4]. Though modifiable, cancers resulting from infectious agents remain a significant burden in LMICs.

Besides the perennial problem of a shortage of pathologists in LMICs, HICs are deploying immunohistochemistry (IHC) and molecular testing to improve diagnostic capabilities, improve prognosis, and allow for individualized treatment decisions (precision medicine). The World Health Organization (WHO) has identified these tests as essential diagnostic tools [[3], [4], [5], [6]]; unfortunately, they are mostly lacking in most LMICs. Inadequate vaccination, cancer screening, and treatment services contribute to the high morbidity and mortality rates from cancer in LMICs [3,5]. Adequate pathology services were available in only a quarter of LMICs in 2017 [6,7]. The WHO has published a guide emphasizing the significance of early cancer diagnosis with emphasis on the evaluation of the disease and subsequent treatment [8]. One of the modifiable risk factors for cancer development is infectious agents. In this article, we review the burden of cancer in LMICs, the common DNA tumor viruses and challenges faced in controlling them in this setting. Although it has not been shown to cause cancer, we also discuss the human polyomaviruses due to the significant morbidity they cause.

## 2 Burden/impact of cancer in LMICs: epidemiological background

According to the International Agency for Research on Cancer (IARC), approximately 20 % of the global population develops cancer at some point in their lives. Furthermore, the statistics indicate that 1 in 8 men and 1 in 11 women succumb to the disease [9]. These recent estimations reveal that over 50 million individuals are currently living within five years of being diagnosed with cancer. The rise in cancer cases can be attributed to factors such as the aging populations worldwide and various socio-economic risk factors. Among women, breast cancer accounts for 25 % of all cancer diagnoses, while colorectal, lung, cervical, and thyroid cancers are also prevalent [10,11]. The WHO projects that these numbers will increase from 20 million to 29.5 million new cancer cases and 16.5 million cancer-related deaths annually by 2040 [12].

While HICs generally have higher cancer incidence rates, the mortality rates and overall number of deaths due to cancer are significantly higher in LMICs, and these rates continue to rise [13]. In 2012, 65 % of all cancer deaths worldwide occurred in LMICs [11,14]. This percentage is projected to increase to 75 % by 2030 [5,9]. There are several reasons for these disparities in cancer trends. HICs have implemented better control measures for risk factors such as infections, anti-smoking campaigns, and other preventive measures [15]. LMICs are facing increasing cancer-related mortality due to factors such as rising obesity rates, sedentary lifestyles, dietary factors, tobacco and alcohol use, and persistent infections like *Helicobacter pylori*, HBV and HPV [16]. LMICs bear a disproportionate burden of infection-associated cancers such as cervical cancer, hepatocellular carcinoma, and gastric cancer [13]. Effective vaccination and eradication therapy for infections like *H. pylori* presents critical opportunities for reducing the global cancer burden [13]. In 2012, approximately 15.4 % of new cancer cases worldwide were attributable to infectious agents [17,18]. This percentage was significantly higher in LMICs compared to HICs, with some countries in sub-Saharan Africa (SSA) having attributable fractions exceeding 50 % [19].

## 3 DNA tumor viruses and human cancers

Several DNA viruses are now known to be oncogenic in the human population, and act as cancer aetiological agents on a global scale. These include HPV, HBV, EBV, Kaposi's sarcoma-associated herpesvirus (KSHV), and human polyomaviruses. Viral infections contribute to an estimated 15 %–20 % of all cancer cases in humans [17]. Through the study of DNA viruses, our understanding of the key molecular factors involved in the transformation process has significantly advanced. Furthermore, research has provided insights into the molecular mechanisms of tumorigenesis employed by these viruses, and there are indications that cofactors may be necessary for viral oncogenicity in certain cases [20].

EBV, HPV, HBV, KSHV, and human polyomaviruses are classified as DNA tumor viruses [21]. They are responsible for causing various types of cancers, such as B cell lymphomas, cervical cancer, liver cancers and head and neck cancers [22]. They achieve this by either expressing viral proteins or ribonucleic acid (RNA) [23]. The viral proteins can activate or suppress host transcription, which ultimately increases oncogene expression or decreases tumor suppressor expression [24]. Proteins encoded by DNA viruses interact with cellular signaling pathways in the host and regulate processes such as cell proliferation, programmed cell death, and genomic integrity [21]. Viral oncoproteins affect several cell signaling pathways, and may be essential for maintaining tumor phenotype. Notable examples include HPV (associated with cervical, anal, oropharyngeal, penile, vulvar and vaginal cancers), EBV (associated with B-cell lymphoproliferative diseases and nasopharyngeal carcinoma), KSHV (associated with Kaposi's Sarcoma and primary effusion lymphomas), and HBV (linked to hepatocellular carcinoma (HCC) [21].

### 3.1 Human papillomavirus (HPVs)

HPVs have a predilection for either cutaneous or mucosal epithelium transmitted through skin-to-skin contact. More than 300 papillomaviruses have been identified and completely sequenced, including over 200 human papillomaviruses [25]. The papillomavirus group has distinct characteristics of genotype-specific-host-restriction and preference of distinct anatomical sites causing clinical pathologies that include benign lesions such as warts, as well as asymptomatic precancer lesions and invasive malignant transformation [26].

HPV belongs to a group of papillomaviruses that are highly specific to certain species and can infect numerous vertebrate species. All papillomaviruses share a similar genome organization and physical structure, consisting of an 8 kb double-stranded, circular DNA viral genome enclosed within a capsid with a diameter of approximately 55 nm. While HPV infection often resolves spontaneously, the lack of an effective immune response can result in the development of genital tract, mouth or head and neck cancers. Unfortunately, there is currently no definitive cure or treatment for all types of HPV-related cancers. However, HPV vaccines are available to prevent infection by the most common types of HPV [27]. A small subset of HPV types can cause cutaneous warts. The approximately 40 types that infect mucosal surfaces are typically spread through sexual contact, including vaginal, anal, or oral sex, and can be divided into low-risk and high-risk types based on their associated cancer risk. Low-risk types cause warts, whereas the 13–14 high-risk types cause cervical intraepithelial neoplasia (CIN) and squamous cell carcinomas (SCC) of the anogenital tract and oropharyngeal mucosa [28].

WHO developed a 3 pillar strategy for eliminating cervical cancer which focuses on primary prevention through vaccination of 90 % of eligible girls by the age of 15 against HPV, secondary prevention through screening 70 % of women with HPV-DNA based tests and treatment of 90 % of women with cervical precancer, and tertiary prevention through timely and appropriate treatment for 90 % of women with invasive cancer. The 3 pillar pathway has the 90-70-90 targets that must be met by 2030 for countries to be on the path towards cervical cancer elimination [29].

#### 3.1.1 Prevention of cervical cancer in LMICs

Prevention is the cornerstone of any effective cervical cancer elimination strategy. The WHO recommends widespread implementation of HPV vaccination to girls between the ages of 9 and 14. In LMICs, significant progress has been achieved in recent years. Through collaboration between governments, WHO, and international partners, several LMICs have successfully introduced HPV vaccination programs. For example, Bhutan and Rwanda were among the first LMICs to introduce HPV vaccination at national levels in 2010 and 2011 respectively and they achieved over 90 % uptake in 12–18 year-old females [30]. Baussano et al. (2021) published data from these two countries which is the first to demonstrate HPV vaccine effectiveness in LMICs [30]. They reported 78 % and 88 % effectiveness against Gardasil vaccine-targeted HPV types in Rwanda and Bhutan respectively [30]. A study conducted in Zimbabwe revealed that the country is vaccinating more than 85 % of eligible girls [31].

#### 3.1.2 Screening methods used in LMICs

Screening for HPV infection is effective in identifying precancerous lesions and allows treatment interventions that can prevent the development of cancer. DNA-based testing for HPV is more effective than today's commonly used screening methods aimed at detecting and preventing cervical cancer [32]. Progress though unacceptably low, has been made in scaling up screening for cervical cancer in LMICs [33]. Various approaches, such as visual inspection with acetic acid (VIA), HPV DNA testing, and cytology (Pap smear), have been utilized. In countries like Malawi, Tanzania, and Nepal, innovative strategies have been implemented, such as mobile clinics, community-based screening, and integration of cervical cancer screening with other healthcare services. These efforts have resulted in increased screening coverage and improved access to services for women in remote areas [[34], [35], [36]].

Though internationally HPV-DNA based cervical cancer screening is now the preferred screening modality, and is a key pillar of the WHO 90-70-90 strategy for the elimination of cervical cancer as a disease of public health concern, it is still expensive and inaccessible to the general population in most LMICs and there is no point of care testing permitting same day treatment. Hence, despite the inferiority of VIA as a screening modality compared to HPV testing and cytology, it remains indispensable in most of these countries for now. The utility of cytology as a screening modality has been hampered by the requirement for cytopathologists and cytotechnologists, scarce resources in LMICs, and long turnaround times for results availability.

#### 3.1.3 Treatment for precancer disease used in LMICs

Treatment options for HPV-related precancer diseases include thermal ablation, cryotherapy, loop electrosurgical excision procedure (LEEP), cold knife conization and in a few selected cases, a simple hysterectomy. Low-technology approaches including VIA screening and treating with cryotherapy or thermal ablation are effective, simple, and widely accepted in LMICs. Over the last decade LMICs who have adopted VIA testing have increased; as of November 2016, 26 countries had incorporated VIA-based screening and treating into national programmes with over 30 using it in pilot programmes [37]. Cryotherapy is a simple and effective treatment modality for cervical intraepithelial neoplasia (CIN) but has some logistical challenges and is mostly being replaced with thermal ablation. LEEP, cold knife conization and simple hysterectomies are more invasive procedures that are used to treat more advanced stages of CIN [28].

Collaborative efforts between WHO, governments, and international partners have resulted in the establishment of specialized cancer centers, training programs for healthcare professionals, and access to essential medicines for the treatment of cervical cancer. Similar initiatives have been implemented in various LMICs, contributing to improved treatment outcomes [38]. However, people still die from this preventable cause because of lack of skilled personnel, infrastructure, radiotherapy equipment and chemotherapeutic agents, lack of finance to continuously visit treatment centers and lack of family and social support. More work still needs to be done to make cervical cancer treatment accessible and affordable to those who need it the most in LMICs.

#### 3.1.4 Challenges in control of cervical cancer LMICs

While some LMICs have successfully implemented HPV vaccination programs, there are still significant challenges in terms of cost, accessibility, and public acceptance. The challenges faced in LMICs include inadequate infrastructure, lack of trained personnel, and limited resources. In addition, there is a lack of awareness about HPV and its associated diseases among the general population, which can lead to low screening rates and late diagnosis of HPV-related diseases [29]. Globally, better treatments for cervical cancer and related diseases are still required. Concurrent chemoradiation remains the mainstay of treatment for disease not amenable to surgical treatment; and targeted therapies being used for advanced and recurrent disease are mostly unavailable in most LMICs.

### 3.2 Hepatitis B virus (HBV)

HBV is double stranded DNA virus belonging to the hepadnavirus family. HBV is endemic globally in the human population, with a number of variants having been described. Apart from HBV, other hepadnaviruses are known to occur in other mammals apart from humans. The disease hepatitis B is a severe liver infection caused by HBV [39]. It can manifest as either a short-term (acute) infection lasting less than six months or a long-term (chronic) infection lasting more than six months [40]. Chronic hepatitis B induces chronic liver inflammation, which can progress to formation of scar tissue (cirrhosis), increasing the risk of liver failure and the potential development of HCC [41]. While most adults with hepatitis B fully recover, even in cases of severe symptoms, infants and children are more susceptible to developing a persistent hepatitis B infection [42]. Although a vaccine is available to prevent hepatitis B, most women are not screened in pregnancy and there is currently no cure for the condition [43].

#### 3.2.1 Screening methods used in LMICs

In LMICs, HBV screening is commonly conducted using rapid diagnostic tests (RDTs) that detect the presence of HBV surface antigen (HBsAg) in blood samples commonly at blood transfusion services, and some antenatal clinics [45]. These tests are cost-effective and user-friendly, although they may not offer the same level of accuracy as laboratory-based tests [45].

#### 3.2.2 Treatment used in LMICs

The treatment of HBV in LMICs typically involves the administration of antiviral medications such as tenofovir and entecavir [46]. These medications aid in suppressing the virus, thereby reducing the risk of liver damage, cirrhosis, and liver cancer [46].

#### 3.2.3 Challenges faced in LMICs

LMICs encounter various challenges in the prevention and treatment of HBV, including limited access to screening and treatment services, insufficient funding, and a lack of public awareness regarding the disease [[44], [45], [46]]. The number of LMICs with national HBV vaccination programs remains limited, with estimates suggesting less than 25 % of low-income countries and less than 30 % of lower-middle income countries having national programs, in sharp contrast to an estimate of 85 % for HICs.

#### 3.2.4 Future perspective of disease burden

WHO has set a target of eliminating viral hepatitis as a public health threat by 2030 [44]. To achieve this objective, the WHO recommends a comprehensive approach that encompasses expanding access to vaccination, screening, and treatment services, as well as enhancing public awareness and political commitment to address the issue [44].

### 3.3 Epstein-Barr virus (EBV)

EBV is a prevalent and highly transmissible viral infection that spreads through saliva and bodily fluids including saliva, blood, and semen [47]. The majority of EBV cases are asymptomatic, but in some instances, particularly among adolescents and young adults, it can result in infectious mononucleosis [47]. EBV belongs to the herpesvirus family and is classified as herpesvirus 4 [47]. Initially, the virus replicates within the epithelial cells of the nasopharynx and subsequently infiltrates B lymphocytes via their CD21 receptors [48]. EBV is linked to several cancer types, including Burkitt's lymphoma, Hodgkin's lymphoma, and nasopharyngeal carcinomas [49]. EBV has the ability to infect B cells and epithelial cells. It can establish a latent infection in B cells, which may contribute to the development of lymphomas [49].

#### 3.3.1 Screening methods used in LMICs

In LMICs, the diagnosis of EBV infection typically relies on clinical symptoms and serological tests [50]. EBV detection and typing through PCR contributes to the diagnostic screening of EBV-positive Burkitt's lymphoma. In a study conducted on childhood B-cell Non-Hodgkin lymphomas (NHL), the detection of EBV was compared using RNA in situ hybridization (RISH) and polymerase chain reaction (PCR). The study findings indicated that PCR assays exhibited a sensitivity of 96 % and specificity of 100 %. Moreover, PCR could detect one EBV genome in 5000–10,000 EBV-negative cells, effectively ruling out the possibility of identifying low-number EBV-bearing memory cells [51].

Another study focused on the detection of low-load EBV in blood samples using droplet digital PCR (ddPCR) [52]. The study compared ddPCR with quantitative PCR (qPCR) and concluded that ddPCR is a highly sensitive and specific method for detecting low-load EBV in blood samples. In terms of sensitivity and accuracy, ddPCR outperformed qPCR [52].

#### 3.3.2 Treatment used in LMICs

The treatment approach for EBV infection in LMICs primarily focuses on supportive care to alleviate symptoms such as fever, sore throat, and fatigue [47]. Antiviral medications like acyclovir and ganciclovir are not widely accessible [50]. Additional research is required to obtain more accurate data on the epidemiology of pediatric NHL in LMICs, as well as to develop treatment protocols that can be safely and effectively implemented in diverse global circumstances [53]. LMICs have limited access to dedicated and comprehensive cancer treatment facilities [54]. T-cell chronic active Epstein-Barr virus (CAEBV) is a rare condition characterized by the predominance of EBV in T cells that infiltrate the tissues, leading to elevated levels of EBV in the blood. If left untreated, patients often experience liver failure, hemophagocytic lymphohistiocytosis, coronary artery aneurysms, impaired organ function due to EBV-infected T cells, or T-cell lymphomas that do not respond to treatment [55]. Currently, hematopoietic stem-cell transplantation is the only curative therapy, emphasizing the importance of an accurate diagnosis and timely initiation of transplantation before the disease reaches an irreversible stage. Specific medications, such as high-dose systemic corticosteroids or a combination of ganciclovir with either histone deacetylase inhibitors or bortezomib, may temporarily alleviate systemic toxicity associated with T-cell CAEBV, allowing the patient to undergo transplantation. Despite transplantation, disease relapses have been observed, and the use of donor-derived virus-specific T cells may offer a potential treatment option for these relapses [56].

#### 3.3.3 Challenges faced in LMICs

LMICs encounter various challenges in managing EBV infection, including limited availability of diagnostic tests, insufficient awareness among healthcare providers, and inadequate treatment resources [50].

#### 3.3.4 Future perspective of disease burden

The future burden of EBV infection is anticipated to rise due to population growth and aging [57]. However, advancements in diagnostic techniques and treatment options hold the potential to mitigate the impact of the disease [57].

### 3.4 Kaposi's sarcoma-associated herpes virus (KSHV)

KSHV is a viral infection that has been linked to the occurrence of three different proliferative diseases: Kaposi sarcoma, primary effusion lymphoma, and multicentric Castleman disease [58]. KSHV is a DNA virus of the herpesviridae family, and is also known as human herpes virus 8 (HHV 8). The identification of this virus took place in 1994, thanks to the work of Chang and Moore [58]. In order for Kaposi sarcoma and primary effusion lymphoma to develop, an individual must be infected with KSHV, which also acts as the causative agent for many cases of multicentric Castleman disease [58]. In Sub-Saharan Africa, KS is among the most common cancer among people living with HIV (PLWH). It can manifest in any immune-compromised person infected with KSHV such as the elderly and transplant recipients. The expression of multiple oncogenic proteins in KSHV leads to the activation of both sequential and parallel signaling pathways, which defines its pathophysiology [58].

Therapeutic approaches have been developed to specifically target these distinct oncogenic proteins encoded by the virus [58].

#### 3.4.1 Screening methods used in LMICs

Screening techniques for KSHV in LMICs encompass serological and molecular methodologies [59]. These approaches are employed to establish an extensive virological profile [59]. Screening facilities in LMICs remain mostly unavailable.

#### 3.4.2 Treatment used in LMICs

The WHO recommends the immediate initiation of highly-active antiretroviral therapy (HAART) for PLWH and Kaposi sarcoma, which is characterized as an AIDS-defining illness. The widespread availability of HAART in LMICs and its early initiation have seen reductions in the incidence of KS; however, it remains a disease of public health importance. Furthermore, the effectiveness of liposomal anthracyclines, like liposomal doxorubicin, has been demonstrated in the treatment of Kaposi sarcoma [58].

#### 3.4.3 Challenges faced in LMICs

LMICs encounter various challenges, such as restricted availability of chemotherapy, insufficiently trained personnel, and inadequate laboratory infrastructure [58].

#### 3.4.4 Future perspective of disease burden

The future outlook for the disease burden of KSHV relies on the ongoing advancement of efficient treatments and the establishment of screening initiatives in LMICs [58].

### 3.5 Human polyomaviruses

Polyomaviruses are a family of viruses that primarily infect mammals and birds [60]. As of 2020, there are 117 species of polyomaviruses, five of which are unassigned to a genus [61]. Most of these viruses are very common and typically asymptomatic in most human populations studied [12]. However, some polyomaviruses are associated with human disease, particularly in immunocompromised individuals [62]. The Polyomaviridae family comprises small circular dsDNA viruses. Among the 14 human polyomaviruses identified to date, BKPyV and JCPyV have been extensively studied since their discovery in 1971 [12]. It has been reported that both BKPyV and JCPyV are widely prevalent worldwide, with a frequency of 80–90 % in various populations [12]. The initial infection caused by these viruses is typically asymptomatic and remains latent until it is activated due to immunosuppression. The activation of BKPyV(BK) and JCPyV(JC) viruses can result in the development of BK Virus Associated Nephropathy and Progressive Multifocal Leukoencephalopathy, respectively. Significant advancements have been made in recent decades in understanding the molecular aspects of polyomaviruses [63].

The BK virus is associated with nephropathy in renal transplant and non-renal solid organ transplant patients [64]. The JC virus is associated with progressive multifocal leukoencephalopathy [65]. The Merkel cell virus is associated with Merkel cell cancer (MCC) [57]. MCC is formerly a rare tumor that has seen a gradual increase in incidence in HICs, where it affects particularly elderly Caucasian males, and occurs mainly in the sun-exposed head and neck areas. This increase in incidence in HICs may be attributable to improvements in diagnostics. However, the epidemiology of MCC in LMICs is not yet described. Probably more attention needs to be paid to the diagnostics in this context. Polyomaviruses are non-enveloped double-stranded DNA viruses with circular genomes of around 5000 base pairs [66]. The genome is packaged in a viral capsid of about 40–50 nm in diameter, which is icosahedral in shape [67]. The capsid is composed of 72 pentameric capsomeres of a protein called VP1, which is capable of self-assembly into a closed icosahedron [67].

JC polyomavirus (JCPyV) is a polyomavirus that specifically infects humans and establishes a lifelong infection in various peripheral organs, primarily those in the urinary tract, in individuals with a healthy immune system [68]. However, in individuals with compromised immune systems, JCPyV can invade the central nervous system (CNS), leading to severe encephalopathies with high morbidity and mortality rates [69]. Liver and bile duct cells can be infected by human polyomaviruses. Moreover, epithelial thymic tumors were found to contain Merkel cell polyomavirus, whereas head and neck carcinomas did not show any detection of this virus [70]. In settings where the immune system is compromised, JCPyV has the ability to invade the central nervous system (CNS), leading to the development of various encephalopathies that have a significant impact on morbidity and mortality. Progressive multifocal leukoencephalopathy (PML), a severe demyelinating brain disease caused by JCPyV, was previously associated with AIDS before the introduction of antiretroviral therapy. However, it has now “re-emerged” as a complication of immunomodulating and chemotherapeutic agents. Currently, there are no effective therapeutics available to specifically target polyomaviruses. The mechanisms by which a depressed immune system allows JCPyV to resurge in the urinary tract, how the virus evades pre-existing antiviral antibodies to become viremic, and the precise route and entry points into the CNS are not fully understood [68].

#### 3.5.1 Screening methods used in LMICs

Polyomaviruses have traditionally been detected and identified using serologic methods, virus isolation by cell culturing, and electron microscopy [71]. However, PCR has recently emerged as a more effective tool for detecting polyomaviruses in clinical samples [66]. Despite its effectiveness, PCR has limitations in routine clinical laboratory implementation due to concerns of contamination and cumbersome detection methods. Real-time PCR instrumentation, such as the LightCycler instrument, has overcome many of these limitations [72]. Some real-time PCR assays use the LightCycler instrument to detect and characterize polyomaviruses. This assay utilizes fluorescence resonance energy transfer (FRET) and two fluorophore-labelled hybridization probes for continuous monitoring of amplicon development [73].

JC virus (JCV) and BK virus (BKV) have the ability to persist in a latent state within the kidneys and can be excreted asymptomatically through urine [74]. JCV has been found to be associated with colorectal and bladder cancers, while BKV has been linked to lung, pancreas, liver, urogenital tract, head, and neck cancers. Hence, it is crucial to assess the presence of JCV DNA and BKV DNA in urine samples from healthy individuals in order to determine their frequency [75].

#### 3.5.2 Treatment used in LMICs

Human polyomavirus is known to cause various diseases in humans, including progressive multifocal leukoencephalopathy (PML) and BK virus-associated nephropathy (BKVN) [76]. Unfortunately, there is currently no specific antiviral treatment available for these infections. However, certain medications like sirolimus and other mechanistic targets of rapamycin inhibitors have demonstrated the ability to directly activate latent pathogenic human polyomavirus replication [77]. It is important to note that the most effective therapy for PML involves reversing the immune-deficient state, as there are no drugs that effectively block virus infection without causing toxicity [65].

#### 3.5.3 Challenges faced in LMICs

Human polyomavirus infections, such as progressive multifocal leukoencephalopathy (PML) and BK virus-associated nephropathy (BKVN), pose a significant health risk in low- and middle-income countries (LMICs) [70]. The diagnosis of these infections is challenging due to the absence of specific symptoms and a reliable diagnostic test. However, the presence of human polyomavirus DNA in cerebrospinal fluid or urine samples can serve as a diagnostic indicator for PML and BKVN, respectively [70]. Regrettably, there is currently no targeted antiviral treatment available for these infections. Nevertheless, certain medications like sirolimus and other mechanistic targets of rapamycin inhibitors have demonstrated the ability to directly activate latent pathogenic human polyomavirus replication [77]. However, the most effective therapy for PML remains the reversal of the immune-deficient state, as there are no drugs that effectively inhibit virus infection without causing toxicity [78]. LMICs face various challenges, including limited access to diagnostic tests, inadequate healthcare infrastructure, and a shortage of trained healthcare professionals [70]. Nonetheless, ongoing efforts are being made to enhance the diagnosis and treatment of human polyomavirus infections in LMICs, which involve the development of new diagnostic tests and the training of healthcare professionals [70].

#### 3.5.4 Future perspective of disease burden

The clinical performance of point-of-care diagnostic tests (POCTs) has been a topic of significant development in recent years, particularly in relation to influenza, RSV, and emerging respiratory viruses. As a result, healthcare professionals, hospital managers, and laboratory directors must regularly update and reassess their best practices. These advancements in diagnostic capabilities may have a profound impact on how we identify, document, and communicate respiratory viral infections in the future [79]. Furthermore, the expectations of patients and healthcare professionals may also change, as patients will likely desire information regarding the responsible pathogen and their clinical prognosis. To ensure the highest quality of care while managing costs, the development of specific antiviral therapies and vaccines will necessitate the creation of new diagnostic algorithms.

In terms of quality of care and clinical management, the future is likely to favor portable, CLIA-waived rapid diagnostic tests that can provide results within 10–20 min. The clinical utility of these tests, or their ability to accurately identify the current cause of a patient's symptoms and differentiate relevant pathogens from bystander pathogens, will be a key criterion for evaluation [80]. Further research is needed to establish correlations between clinical outcomes and laboratory data. Additionally, the timing of diagnostic testing in relation to a patient's illness will be a crucial consideration. The sensitivity and positive predictive value of a diagnostic test depend on factors such as specimen quality, virus load (which is typically higher in children and early in the course of illness), duration of viral shedding, and the patient's immune status. Future diagnostic algorithms must take these factors into account, as well as the epidemiology of viruses in a given season or region.

In addition to the acknowledged species, there have been reports of PyVs genomes that have not yet been classified as species. It is likely that some of these genomes will be assigned as species in the future [81]. Biosensors offer a reliable and cost-effective method for detecting specific pathogens in point-of-care settings. Recently, various types of sensors have been developed for the rapid identification of respiratory viruses. Layqah and Eissa (2019) were able to detect MERS-CoV spike protein within 20 min using a gold-coated array of carbon electrodes [82]. This electrochemical assay relies on the competitive binding of a MERS-CoV antibody to either the virus in the sample or the immobilized antigen on the electrode. The reduced peak current through the chip can then be measured. In theory, this technique can be expanded to simultaneously detect multiple viruses, but its diagnostic performance needs to be validated using patient samples [82]. Another sensitive technique is based on surface plasmon resonance (SPR), where biomolecules bound to a metal surface cause a reduction in the reflection of incident light [83]. Using a new antibody against a recombinant AIV H7N9, the authors in one study achieved a detection limit of a few hundred copies per mL nasal fluid within 10 min [84]. Although still in the experimental stage, the characteristics of this approach make it a promising candidate for future rapid point-of-care testing [84].

## 4 Conclusion

It is currently estimated that around 15–20 % of cancers worldwide can be attributed to viral infections. The medical community has shown great interest in studying the impact of DNA tumor viruses in LMICs. The literature revealed that approximately 85 % of virus-induced cancers occur in developing countries. Different mechanisms are employed by oncogenic viruses to induce cancer. However, it is crucial to acknowledge that only a minority of individuals who are infected actually develop cancer, and this typically occurs after years of chronic infection. The viruses most commonly associated with cancer cases are the HPVs, particularly in relation to cervical cancer and other epithelial malignancies. Additionally, the hepatitis viruses HBV and HCV significantly contribute to hepatocellular cancer. Other oncoviruses include EBV, KSHV, human T-cell leukemia virus (HTLV-I), and Merkel cell polyomavirus (MCPyV). The identification of these infectious agents responsible for cancer has led to various interventions aimed at reducing the risk of developing these diseases. These interventions include preventive vaccines for HBV and HPV, HPV-based testing for cervical cancer screening, antiviral treatments for chronic HBV and HCV infections, and screening of the blood supply for HBV and HCV. Successful efforts in identifying additional oncogenic viruses in human cancer may provide further insights into the causes and development of cancer, as well as new approaches for prevention and treatment. The impact of DNA tumor viruses on LMICs requires a multidisciplinary approach. Further research is necessary to gain a deeper understanding of the mechanisms behind virus-induced cancers and to develop effective prevention and treatment strategies.

## CRediT authorship contribution statement

**Bothwell Takaingofa Guzha:** Writing – review & editing, Writing – original draft, Conceptualization. **Allen Matubu:** Writing – review & editing, Writing – original draft. **George Nyandoro:** Writing – review & editing, Writing – original draft. **Hamish O. Mubata:** Writing – review & editing, Writing – original draft. **Enos Moyo:** Writing – review & editing. **Grant Murewanhema:** Writing – review & editing, Supervision. **Zvavahera M. Chirenje:** Writing – review & editing, Resources, Project administration, Conceptualization.

## Declaration of competing interest

The authors declare that they have no known competing financial interests or personal relationships that could have appeared to influence the work reported in this paper.

## Data Availability

No data was used for the research described in the article.

## References

[1] Purushothaman P., Uppal T., Verma S. (2020). Human DNA tumor viruses and oncogenesis. Anim. Biotechnol..

[2] Howley P., Livingston D. (2009). Small DNA tumor viruses: large contributors to biomedical sciences. Virology.

[3] Radich J.P., Briercheck E., Chiu D.T., Menon M.P., Sala Torra O., Yeung C.C. (2022). Precision medicine in low-and middle-income countries. Annu. Rev. Pathol..

[4] Drake T., Knight S., Harrison E., Søreide K. (2018). Global inequities in precision medicine and molecular cancer research. Front. Oncol..

[5] The Lancet (2018). Globocan 2018: counting the toll of cancer. Lancet.

[6] World Health Organization (2019). Second WHO model list of essential in vitro diagnostics. https://www.who.int/publications/i/item/WHO-MVP-EMP-2019.05.

[7] Gultekin M., Ramirez P., Broutet N., Hutubessy R. (2020). World Health Organization call for action to eliminate cervical cancer globally. Int. J. Gynecol. Cancer.

[8] da Silva R. (2019). Guide to cancer early diagnosis. Rev Bras Cancerol.

[9] International Agency for Research on Cancer (2018). Global cancer observatory. https://gco.iarc.fr/en.

[10] Bray F., Ferlay J., Soerjomataram I., Siegel R.L., Torre L.A., Jemal A. (2018). Global cancer statistics 2018: GLOBOCAN estimates of incidence and mortality worldwide for 36 cancers in 185 countries. CA A Cancer J. Clin..

[11] Ferlay J., Ervik M., Lam F., Colombet M., Mery L., Piñeros M. (2020). Cancer Today.

[12] Hussain I., Tasneem F., Gilani U.S., Arshad M.I., Farhan Ul, Haque M. (2020). Human BK and JC polyomaviruses: molecular insights and prevalence in Asia. Virus Res..

[13] Torre L., Siegel R., Ward E., Jemal A. (2016). Global cancer incidence and mortality rates and trends-an update. Cancer Epidemiol. Biomarkers Prev..

[14] Parkin D., Bray F., Ferlay J., Pisani P. (2005). Global cancer statistics, 2002. CA A Cancer J. Clin..

[15] Akinyemiju T., Ogunsina K., Gupta A., Liu I., Braithwaite D., Hiatt R.A. (2022). A socio-ecological framework for cancer prevention in low and middle-income countries. Front. Public Health.

[16] Herchenhorn D., Freire V. (2022). Access of new systemic therapies for Genito-urinary cancers in low-middle income countries. Front Urol.

[17] Ahmed W., Zaib S., Ullah S., Fatima A., Zaib Z., Haseeb Azam M.A. (2023). Role of human papillomavirus in various cancers: epidemiology, screening and prevention. Mini Rev. Med. Chem..

[18] Talukdar A., Kataki A., Banavali S., Ghosh J., Kataki A., Barmon D. (2023). Fundamentals in Gynaecologic Malignancy.

[19] Collatuzzo G., Boffetta P. (2023). Cancers attributable to modifiable risk factors: a road map for prevention. Annu. Rev. Publ. Health.

[20] Damania B. (2007). DNA tumor viruses and human cancer. Trends Microbiol..

[21] Fazlalipour M., Ghoreshi Z., Molaei H., Arefinia N. (2023). The role of DNA viruses in human cancer. Cancer Inf..

[22] Mestiri S., El-Ella D.M., Fernandes Q., Bedhiafi T., Almoghrabi S., Akbar S. (2024). The dynamic role of immune checkpoint molecules in diagnosis, prognosis, and treatment of head and neck cancers. Biomed. Pharmacother..

[23] Spurgeon M. (2022). Small DNA tumor viruses and human cancer: preclinical models of virus infection and disease. Tumour Virus Res..

[24] Ameya G., Birri D. (2023). The molecular mechanisms of virus-induced human cancers. Microb. Pathog..

[25] National Institute of Allergy and Infectious Diseases. The Papillomavirus Episteme. n.d. Available at: https://pave.niaid.nih.gov/(Accessed: 15 January 2024).

[26] Bottalico D., Chen Z., Dunne A., Ostoloza J., McKinney S., Sun C. (2011). The oral cavity contains abundant known and novel human papillomaviruses from the Betapapillomavirus and Gammapapillomavirus genera. J. Infect. Dis..

[27] Tripathi A., Sahu U., Khare P., Jain A. (2022). Developments in Microbiology, Immunopathology, Diagnosis and Treatment of HPV Induced Malignancies.

[28] Quinlan J. (2021). Human papillomavirus: screening, testing, and prevention. Am. Fam. Physician.

[29] World Health Organization (2020). Global strategy to accelerate the elimination of cervical cancer as a public health problem. https://www.who.int/publications/i/item/9789240014107.

[30] Baussano I., Sayinzoga F., Tshomo U., Tenet V., Vorsters A., Heideman D.A. (2021). Impact of human papillomavirus vaccination, Rwanda and Bhutan. Emerg. Infect. Dis..

[31] Tapera O., Nyakabau A.M., Simango N., Guzha B.T., Jombo-Nyakuwa S., Takawira E. (2021). Gaps and opportunities for cervical cancer prevention, diagnosis, treatment and care: evidence from midterm review of the Zimbabwe cervical Cancer prevention and control strategy (2016-2020). BMC Publ. Health.

[32] Williams J., Kostiuk M., Biron V. (2022). Molecular detection methods in HPV-related cancers. Front. Oncol..

[33] Lemp J.M., De Neve J.W., Bussmann H., Chen S., Manne-Goehler J., Theilmann M. (2020). Lifetime prevalence of cervical cancer screening in 55 low- and middle-income countries. JAMA.

[34] Darj E., Chalise P., Shakya S. (2019). Barriers and facilitators to cervical cancer screening in Nepal: a qualitative study. Sex Reprod Healthc.

[35] Msyamboza K.P., Phiri T., Sichali W., Kwenda W., Kachale F. (2016). Cervical cancer screening uptake and challenges in Malawi from 2011 to 2015: retrospective cohort study. BMC Publ. Health.

[36] PATH (2022). Global HPV vaccine introduction overview. https://www.path.org/our-impact/resources/global-hpv-vaccine-introduction-overview/.

[37] Cubie H., Campbell C., Weller D. (2017). Tackling the burden of cervical cancer: lessons from Malawi and other low- and middle-income countries. https://www.cancercontrol.info/2017-4/tackling-the-burden-of-cervical-cancer-lessons-from-malawi-and-other-low-and-middle-income-countries/.

[38] Maza M., Schocken C.M., Bergman K.L., Randall T.C., Cremer M.L. (2016). Cervical precancer treatment in low- and middle-income countries: a technology overview. J Glob Oncol.

[39] Tsukuda S., Watashi K. (2020). Hepatitis B virus biology and life cycle. Antivir. Res..

[40] Choi H.S., van Campenhout M.J., van Vuuren A.J., Krassenburg L.A., Sonneveld M.J., de Knegt R.J. (2021). Ultra-Long-term follow-up of interferon alfa treatment for HBeAg-positive chronic hepatitis B virus infection. Clin. Gastroenterol. Hepatol..

[41] Kadiri D., Peela S., Ganguli D., Nagaraju G.P., Peela S. (2022). Theranostics and Precision Medicine for the Management of Hepatocellular Carcinoma.

[42] Hefele L., Vannachone S., Khounvisith V., Nouanthong P., Sayasone S., Kounnavong S. (2020). Lasting benefit of infant hepatitis B vaccination in adolescents in the Lao People's Democratic Republic. Int. J. Infect. Dis..

[43] Pattyn J., Hendrickx G., Vorsters A., Van Damme P. (2021). Hepatitis B vaccines. J. Infect. Dis..

[44] World Health Organization (2021). Interim guidance for country validation of viral hepatitis elimination. https://www.who.int/publications/i/item/9789240028395.

[45] Shenge J., Osiowy C. (2021). Rapid diagnostics for hepatitis B and C viruses in low-and middle-income countries. Front Virol.

[46] Wilkins T., Sams R., Carpenter M. (2019). Hepatitis B: screening, prevention, diagnosis, and treatment. Am. Fam. Physician.

[47] Arai A. (2021). Chronic active epstein-barr virus infection: the elucidation of the pathophysiology and the development of therapeutic methods. Microorganisms.

[48] Syrykh C., Péricart S., Lamaison C., Escudié F., Brousset P., Laurent C. (2021). Epstein-barr virus-associated T- and NK-cell lymphoproliferative diseases: a review of clinical and pathological features. Cancers.

[49] Lee O.Z., Omar N., Tay J., Lee V. (2023). A clinicopathology review and update of epstein-barr virus-associated mesenchymal tumors. Cancers.

[50] Soldan S., Lieberman P. (2023). Epstein-Barr virus and multiple sclerosis. Nat. Rev. Microbiol..

[51] Hassan R., White L.R., Stefanoff C.G., de Oliveira D.E., Felisbino F.E., Klumb C.E. (2006). Epstein-Barr virus (EBV) detection and typing by PCR: a contribution to diagnostic screening of EBV-positive Burkitt's lymphoma. Diagn. Pathol..

[52] Li J.Y., Chen X.P., Tie Y.Q., Sun X.L., Zhang R.Q., He A.N. (2022). Detection of low-load Epstein-Barr virus in blood samples by enriched recombinase aided amplification assay. Amb. Express.

[53] Ozuah N., El-Mallawany N. (2019). Abla A Non-hodgkin's Lymphoma in Childhood and Adolescence.

[54] Sirohi B., Chalkidou K., Pramesh C.S., Anderson B.O., Loeher P., El Dewachi O. (2018). Developing institutions for cancer care in low-income and middle-income countries: from cancer units to comprehensive cancer centres. Lancet Oncol..

[55] Bollard C., Cohen J. (2018). How I treat T-cell chronic active Epstein-Barr virus disease. Blood.

[56] Fałkowska A., Prądzyńska K., Drabko K. (2021). Difficult balance between EBV treatment and posttransplant immunosuppression: a successful transplant in a child with recurrent Epstein-barr virus-induced hemophagocytic lymphohistiocytosis. Transplant. Proc..

[57] Yang Y., Gao F. (2020). Clinical characteristics of primary and reactivated Epstein-Barr virus infection in children. J. Med. Virol..

[58] Sullivan R.J., Pantanowitz L., Casper C., Stebbing J., Dezube B.J. (2008). Epidemiology, pathophysiology, and treatment of Kaposi sarcoma-associated herpesvirus disease: Kaposi sarcoma, primary effusion lymphoma, and multicentric Castleman disease. Clin. Infect. Dis..

[59] Zhang X., Xu Y., Li Y., Yuan H., Liu Z., Zhang T. (2022). Prevalence and correlates of Kaposi's sarcoma-associated herpesvirus and herpes simplex virus type 2 infections among adults: evidence from the NHANES III data. Virol. J..

[60] Fehér E., Kaszab E., Mótyán J.A., Máté D., Bali K., Hoitsy M. (2024). Structural similarity of human papillomavirus E4 and polyomaviral VP4 exhibited by genomic analysis of the common kestrel (Falco tinnunculus) polyomavirus. Vet. Res. Commun..

[61] Lazim H., Al-Obaidi A. (2023). Wu and KI polyomaviruses. Microbes Infect Dis.

[62] Ciotti M., Prezioso C., Pietropaolo V. (2019). An overview on human polyomaviruses biology and related diseases. Future Virol..

[63] Harprecht C. (2020). Structural analysis of JC polyomavirus inhibitors and receptors. Tübingen: Universität Tübingen.

[64] Kuppachi S., Kaur D., Holanda D., Thomas C. (2016). BK polyoma virus infection and renal disease in non-renal solid organ transplantation. Clin Kidney J.

[65] Cortese I., Reich D., Nath A. (2021). Progressive multifocal leukoencephalopathy and the spectrum of JC virus-related disease. Nat. Rev. Neurol..

[66] Furmaga J., Kowalczyk M., Zapolski T., Furmaga O., Krakowski L., Rudzki G. (2021). BK polyomavirus-biology, genomic variation and diagnosis. Viruses.

[67] van Rosmalen M. (2018).

[68] Butic A., Spencer S., Shaheen S., Lukacher A. (2023). Polyomavirus wakes up and chooses neurovirulence. Viruses.

[69] Kaiserman J., O'Hara B., Haley S., Atwood W. (2023). An elusive target: inhibitors of JC polyomavirus infection and their development as therapeutics for the treatment of progressive multifocal leukoencephalopathy. Int. J. Mol. Sci..

[70] Klufah F., Mobaraki G., Liu D., Alharbi R.A., Kurz A.K., Speel E.J. (2021). Emerging role of human polyomaviruses 6 and 7 in human cancers. Infect. Agents Cancer.

[71] Giraldo G., Beth E., Lee J., de Harven E., Chernesky M. (1982). Solid-phase immune electron microscopy-double-antibody technique for rapid detection of papovaviruses. J. Clin. Microbiol..

[72] Gorgannezhad M. (2020).

[73] Pham L. (2012).

[74] Alalawi F., Alnour H., El Kossi M., Jenkins J., Taku A., Sharma A.K. (2020). BK virus infection in renal transplant recipients: an overview. J Egypt Soc Nephrol Transplant.

[75] Karimi Dehcheshmeh L., Makvandi A. (2020). Prevalence of human polyomavirus JC and BK in normal population. Asian Pac. J. Cancer Prev. APJCP.

[76] Lim F.L., Teo A.L., Loh Y.S., Hwang W.Y., Goh Y.T., Ling K.L. (2011). Evaluation of human polyomavirus (BKV)–Specific T cell immune reconstitution in allogeneic haematopoietic stem cell transplant recipients. Blood.

[77] Alvarez Orellana J., Kwun H.J., Artusi S., Chang Y., Moore P.S. (2021). Sirolimus and other mechanistic target of rapamycin inhibitors directly activate latent pathogenic human polyomavirus replication. J. Infect. Dis..

[78] Ariav Y., Ch'ng J.H., Christofk H.R., Ron-Harel N., Erez A. (2021). Targeting nucleotide metabolism as the nexus of viral infections, cancer, and the immune response. Sci. Adv..

[79] Nelson A., Milner D., Rebbeck T., Iliyasu Y. (2016). Oncologic care and pathology resources in Africa: survey and recommendations. J. Clin. Oncol..

[80] Neal R.D., Tharmanathan P., France B., Din N.U., Cotton S., Fallon-Ferguson J. (2015). Is increased time to diagnosis and treatment in symptomatic cancer associated with poorer outcomes? Systematic review. Br. J. Cancer.

[81] Torres C. (2020). Evolution and molecular epidemiology of polyomaviruses. Infect. Genet. Evol..

[82] Layqah L., Eissa S. (2019). An electrochemical immunosensor for the corona virus associated with the Middle East respiratory syndrome using an array of gold nanoparticle-modified carbon electrodes. Microchim. Acta.

[83] Nguyen H., Park J., Kang S., Kim M. (2015). Surface plasmon resonance: a versatile technique for biosensor applications. Sensors.

[84] Chang P., Lukosaityte D., Sealy J.E., Rijal P., Sadeyen J.R., Bhat S. (2023). Antigenic characterization of human monoclonal antibodies for therapeutic use against H7N9 avian influenza virus. J. Virol..

